# The roles of tumor-derived exosomes in altered differentiation, maturation and function of dendritic cells

**DOI:** 10.1186/s12943-021-01376-w

**Published:** 2021-06-02

**Authors:** Reza Hosseini, Leila Asef-Kabiri, Hassan Yousefi, Hamzeh Sarvnaz, Majid Salehi, Mohammad Esmaeil Akbari, Nahid Eskandari

**Affiliations:** 1 Department of Immunology, School of Medicine, Isfahan University of Medical Sciences, Isfahan, Iran; 2 Cancer Research Center, Shahid Beheshti University of Medical Sciences, Tehran, Iran; 3 Department of Biochemistry and Molecular Biology, LSUHSC School of Medicine, New Orleans, USA; 4 Department of Immunology, School of Public Health, Tehran University of Medical Sciences, Tehran, Iran; 5 Department of Tissue Engineering, School of Medicine, Shahroud University of Medical Sciences, Shahroud, Iran

**Keywords:** Exosome, Tumor, Dendritic cell, Immunity

## Abstract

Tumor-derived exosomes (TDEs) have been shown to impede anti-tumor immune responses via their immunosuppressive cargo. Since dendritic cells (DCs) are the key mediators of priming and maintenance of T cell-mediated responses; thus it is logical that the exosomes released by tumor cells can exert a dominant influence on DCs biology. This paper intends to provide a mechanistic insight into the TDEs-mediated DCs abnormalities in the tumor context. More importantly, we discuss extensively how tumor exosomes induce subversion of DCs differentiation, maturation and function in separate sections. We also briefly describe the importance of TDEs at therapeutic level to help guide future treatment options, in particular DC-based vaccination strategy, and review advances in the design and discovery of exosome inhibitors. Understanding the exosomal content and the pathways by which TDEs are responsible for immune evasion may help to revise treatment rationales and devise novel therapeutic approaches to overcome the hurdles in cancer treatment.

## Introduction

Exosomes are nano-sized (30–150 nm) extracellular vesicles released virtually by all types of cells and their content robustly mirrors that of the parental cells [1]. In particular, tumor cells were shown to actively secrete a large amount of exosomes to provide intercellular communication with surrounding as well as distant cells [1]. These extracellular vesicles contain several types of mRNAs, micro RNAs, functional surface proteins, enzymes and lipids, which enable them to exert local or systemic effects through direct interactions with the cell surface receptors or via transferring their contents into recipient cells through plasma membrane fusion, endocytosis, phagocytosis, micro pinocytosis, and lipid raft-mediated internalization [2, 3]. Compelling evidence demonstrates that tumor-derived exosomes (TDEs) function in favor of tumor progression and crucially participate in nearly all aspects of cancer development, such as angiogenesis, proliferation, and metastasis [2].

In addition, TDEs also give an advantage to tumor outgrowth by negatively regulating anti-cancer immune responses [4]. Several studies have shown that TDEs could inhibit anti-tumor immunity either through internalization by the target cells or through receptor-ligand interactions [5, 6]. In this regard, it has been acknowledged that TDEs harbor a plethora of membrane-bound proteins (Fas-L, PD-L1, etc.) that can directly inhibit the anti-tumor activity of effector CD8+ T cells and NK cells [7]. More importantly, on the other hand, exosomes released from tumor cells can also be taken up or interact with antigen presenting cells (APCs) and may indirectly induce antigen-specific tolerance [8]. Of particular note, TDEs especially target dendritic cells (DCs) which are the most important and effective APCs that orchestrate immune responses by priming naive T cells and providing subsequent signals required for the activity of effector T cells [8]. In this regard, it has been shown that TDEs largely inhibit the differentiation of DCs from bone marrow progenitors and monocytes, while strongly promote the development of tumor supportive cells, such as myeloid-derived suppressor cells (MDSCs) [9–11]. Tumor-derived exosomes were also shown to carry several bioactive molecules that can interfere with the maturation of DCs, thus demolishing their capability in inducing effective anti-tumor responses [12]. Moreover, others have shown that TDEs can alter the function of well-differentiated mature DCs. According to the published data, the interaction/uptake of TDEs by mature DCs renders them to an immunosuppressive phenotype, which thereby can improve tumor immune evasion [13, 14].

On the contrast, since TDEs contain a variety of tumor-associated antigens, there is a large degree of consensus that exosomes released by cancer cells can stimulate DCs to support potent anti-tumor immunity development [15]. However, growing evidence indicates that the dominant effect of TDEs is immunosuppression, rather than immunostimulation [16]. Taken together, TDEs seems to negatively affect DCs, as the key mediators of immune responses, to prevent the development of effective anti-tumor immunity. However, a literature review on the molecular mechanisms by which tumor-derived exosomes interfere with the biology of DCs is still lacking. Therefore, in the present study, we provide the published evidence on how TDEs could impair the differentiation, maturation, and function of DCs. We then briefly discuss the lessons learned from TDEs-mediated DCs abnormalities for the translation of research into practice, and review advances in the design and development of exosome inhibitors as potential adjunctive therapy for cancer.

### Tumor-derived exosomes alter differentiation of DCs

Dendritic cells (DCs) are rare types of immune cells that differentiate from both myeloid and lymphoid progenitors in the bone marrow or derive from monocytic cells, and are largely localized in tissues [17]. Several subgroups of DCs have been identified, but plasmacytoid DCs (pDCs) and conventional DCs (cDCs) are the most common populations. Plasmacytoid DCs mainly produce type I interferons, however the latters are key Ag presenting cells (APCs) optimally initiate naive/resting T cell responses [18]. Because of their specialized characteristics, cDCs actively capture, internalize, and process the foreign pathogenic Ags and self-non-tumor or tumor-derived Ags and then present to CD4+ and CD8+ T cells via the MHC-II and MHC-I molecules, respectively [18]. It is now evident that the abnormal differentiation of DCs is one of the main contributors of non-responsiveness to tumors [19, 20]. The impaired differentiation of DCs in the tumor context has been highlighted with the dominant infiltration of myeloid-derived suppressor cells (MDSCs) and decreased number/accumulation of mature DCs in several malignancies including breast, lung, cervical, and colorectal tumors [21]. Additionally, clear evidence indicates that the defects of DCs in cancers are systemic rather than localized to the tumor sites [22]. These observations imply that the tumor-derived soluble factors might potentially play a major role in the defective differentiation of DCs in the tumor context [23]. Several factors derived from tumors as well as associated cells from the surrounding tumor microenvironment (TME) have been described to interfere with DCs differentiation. However, growing data have emphasized the role of tumor-derived exosomes (TDEs) in the loss of stimulatory APC activity and subsequently diminished anti-tumor immune responses in tumor-bearing hosts [11]. Here, we summarized the published data on the mechanisms by which TDEs could alter the differentiation of DCs in tumors.

Early studies have shown that the administration of TDEs considerably increases a population of undifferentiated myeloid progenitors [24]. Indeed, an increment of myeloid-derived suppressor cells (MDSCs) is the hallmark of defects in DCs differentiation [25]. Several lines of evidence have indicated that TDEs can corrupt myelopoiesis in the cancer by blocking the differentiation of myeloid precursors (including DCs precursors), which results in fewer DCs and an accumulation of myeloid cells with immunosuppressive function called MDSCs [21]. The molecular mechanisms that drive this process are not completely understood and various biomolecules are assumed to be involved in the TDEs-mediated accumulation of MDSCs. Previous studies have shown that prostanoids (i.e. PGE2) derived from cyclooxygenase-1 (COX-1) and COX-2 can inhibit the differentiation of both bone marrow- and monocyte-derived DCs [26, 27]. Tumor-derived exosomes have also been shown to carry functional COX-2 enzymes and its product, PGE2 [16, 28, 29]. It was demonstrated that the internalization of TDEs containing PGE2 and TGF-β by bone marrow precursors impedes DCs differentiation and instead promotes the induction of MDSCs [30, 31]. However, targeting exosomal PGE2 and TGF-β abolished the ability of TDEs to induce MDSCs and restored DCs differentiation, indicating their pivotal role in DCs abnormalities [30, 31]. Moreover, it was shown that COX-2 can be exported via TDEs into target cells, which may further increase PGE2 secretion in TME and promote tumor growth [28, 32]. Tumor-derived exosomes were also reported to induce MDSCs through STAT-3 dependent manner [33]. Multiple evidence shows that TDEs release considerable amounts of IL-6, a well-known STAT-3 activator, which has widely been recognized to inhibit DCs differentiation from CD34+ bone marrow progenitors [18, 33, 34]. Additionally, IL-6 released from TDEs has also been found to promote proliferation and inhibit apoptosis of MDSCs [21, 30, 35]. Likewise, it was demonstrated that exosomes derived from TS/A murine mammary tumor cells target human monocytes and myeloid precursors of the bone marrow and block their differentiation into DCs, mainly via IL-6 and STAT3 pathways [11]. Tumor-derived exosomes were also shown to contain several other activating components of the STAT-3 pathway, including HSP70 and HSP72, which can induce the development of MDSCs [36, 37]. Nevertheless, other intracellular pathways might also be involved in TDEs-mediated DCs abnormal differentiation. In this regard, it has been shown that melanoma-derived exosomes can inhibit the differentiation of DCs from bone marrow progenitors with wild-type MyD88; however, no inhibitory effect was observed in MyD88-deficient precursors, demonstrating that TDEs can exploit the MyD88 pathway for preventing DCs differentiation [38].

**Fig. 1:**
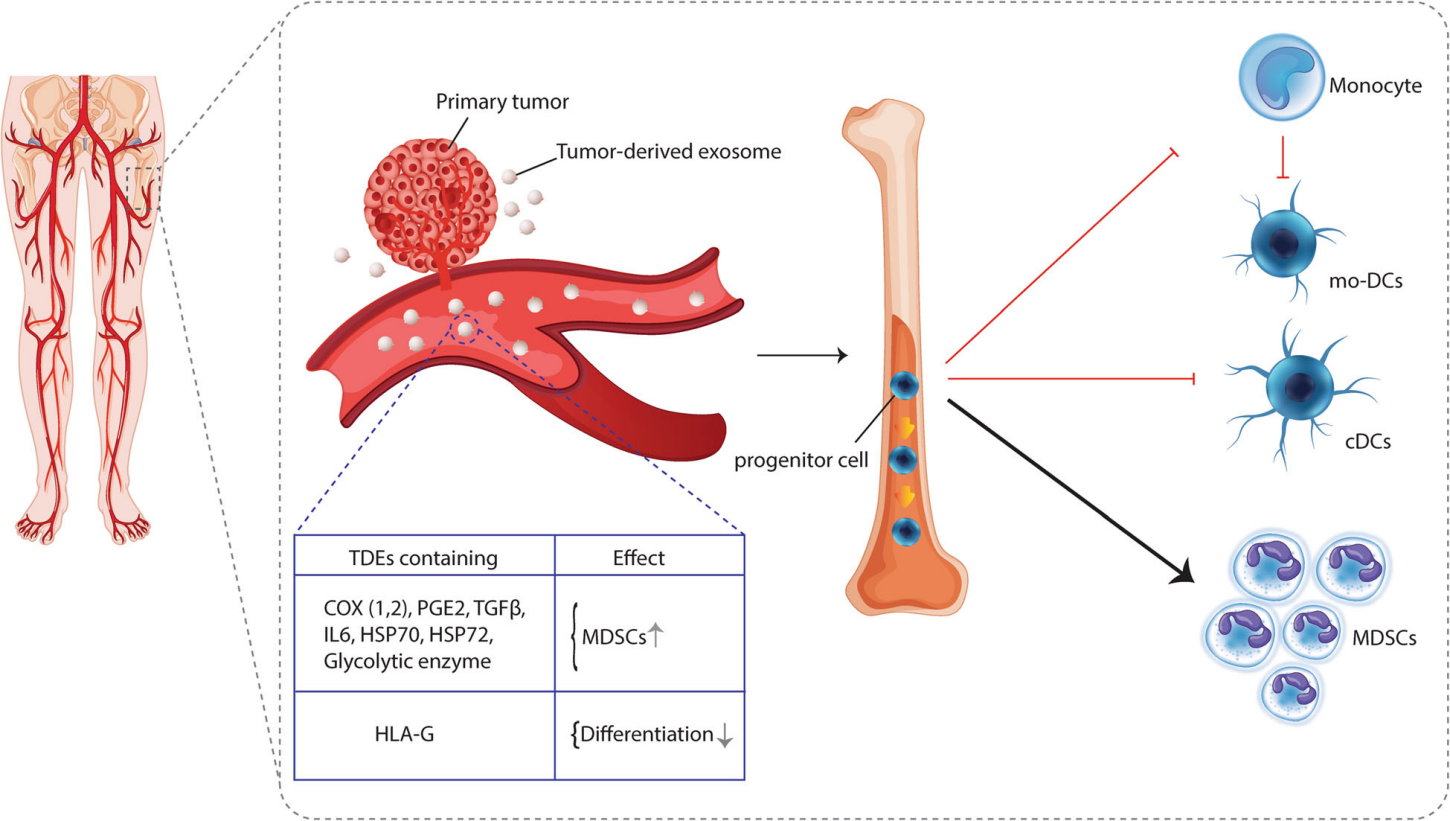
Tumor-derived exosomes inhibit differentiation of dendritic cells. Tumor-derived exosomes contain several biomolecules including COX-2 (cyclooxygenase-2), PGE2 (prostaglandin E2), TGF-β (transforming growth factor- β), IL-6, HSP70, HSP72, HLA-G and glycolytic enzymes, thereby could affect bone marrow progenitors and inhibit differentiation of DCs and monocytes, while promoting the polarization of myeloid-derived suppressor cells (MDSCs). Exosomes derived from tumors can also impede monocytes differentiation toward DCs. Mo-DCs: monocyte-derived dendritic cells

More recent data show that human leukocyte antigen G (HLA-G) molecules are also expressed on TDEs and play a key role in inhibiting DCs differentiation [39]. HLA-G is a non-classical MCH-I molecule that aberrantly expressed in a variety of human tumors and mediates suppression of T cells, NK cells and DCs through binding to inhibitory receptors [40, 41]. It has been found that cancer stem cell (CSC)-derived exosomes bearing HLA-G can inhibit monocyte-derived DCs differentiation [39]. However, blocking HLA-G with antibodies nullified the effects of CSC-derived exosomes on DCs differentiation suggesting that HLA-G carried by extracellular vesicles plays an immunomodulatory role [39] (Fig. 1). Additionally, tumor exosomes are assumed to inhibit the differentiation of DCs through metabolic reprogramming [18]. Of note, TDEs are widely enriched in glycolytic enzymes converting glucose into extracelullar ATP and lactate in the local tumor-microenvironment [42, 43]. The accumulation of lactic acid can restrain the differentiation of DCs, whereas promoting the expansion of myeloid-derived suppressor cells (MDSCs) [44, 45] (Fig. 1).

### Tumor-derived exosomes alter maturation of DCs

Under the normal conditions, DCs are in an immature state expressing higher levels of phagocytic receptors, while characterized by low antigen-presenting capabilities [46]. Upon being induced by pathogen-associated molecular patterns (PAMPs) or damage-associated molecular patterns (DAMPs) through receptors such as CD40, TNF-R, IL-1R, and TLRs, DCs acquire a mature state expressing higher levels of antigen-loaded MHC-I and MHC-II molecules as well as costimulatory signaling B7 family molecules (e.g., CD80 and CD86) [46]. The presence of mature tumor-infiltrating DCs has been linked with the magnitude of anti-tumor T cell responses and a better prognosis in cancer patients [47]. However, in the context of tumors, DCs are mainly found in an immature phenotype unable to support normal levels of antigen-specific T cell expansion, leading to the induction of peripheral tolerance [48]. It is often unclear whether the immature phenotype of DCs reflects a simple failure of tumors to support the maturation and activation of these cells or, alternatively, active suppression of DCs maturation by tumors [26]. Up to now, several attempts have been made to resolve the intricacies dampening tumor-associated DCs maturation; but the limiting number of DCs that can be isolated from tumor-bearing animals and cancer patients and the complex nature of the cells and soluble factors present within the TME have made it difficult to gain mechanistic insights into the tumor-associated-impaired DC maturation in vivo [26]. In this regard, monocytic- and bone marrow-derived DCs (BMDCs) have been employed as suitable alternative ex vivo models to study the defective maturation of DCs by tumor cells or tumor-derived soluble factors [49]. The most recent studies, summarized in the following section, suggest that TDEs harboring several immunosuppressive biomolecules actively participate in the impaired maturation of DCs [12, 50].

**Fig. 2:**
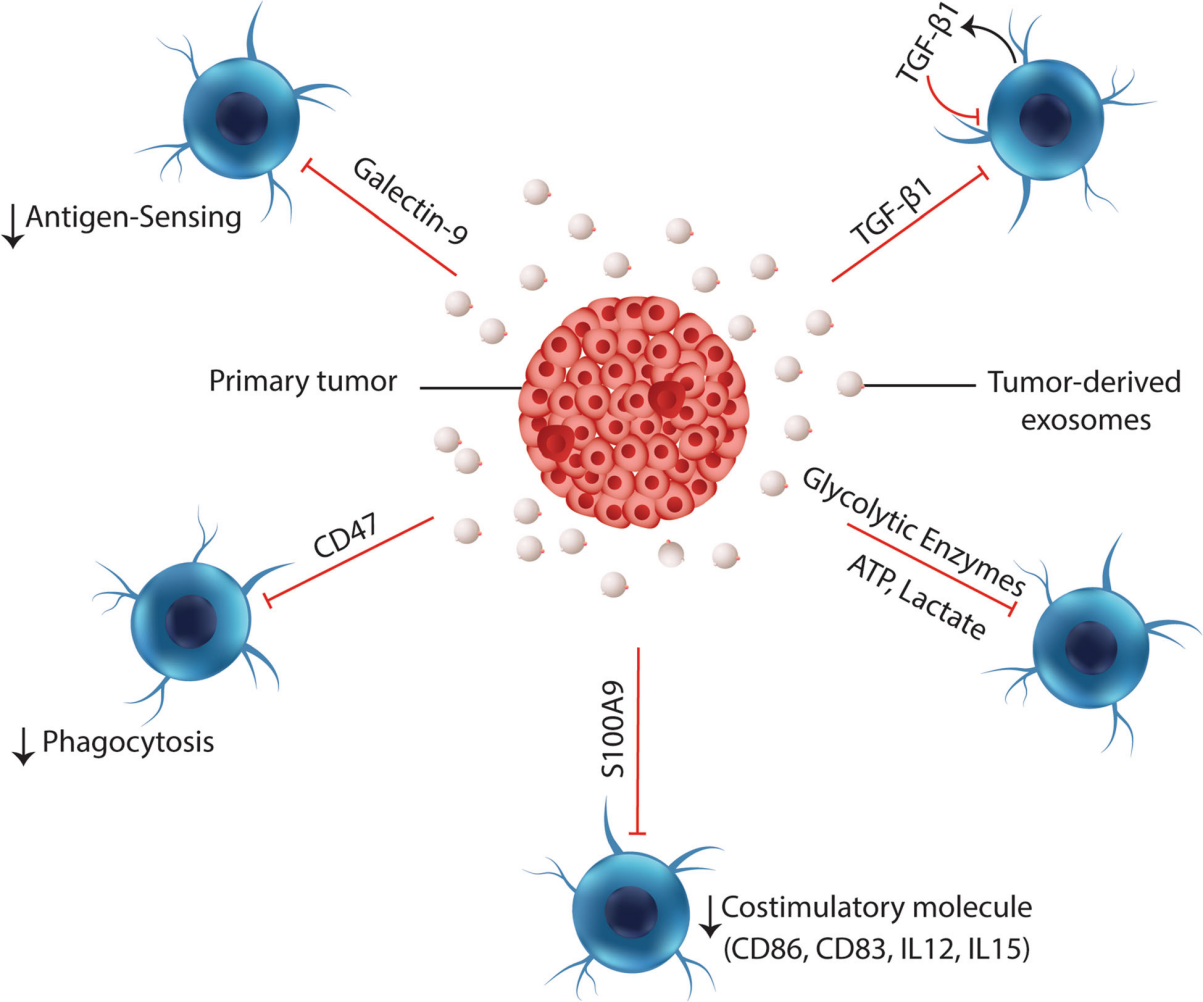
Tumor-derived exosomes inhibit maturation of dendritic cells. Exosomal galectin-9 can interact with its cognate TIM-3 receptors on DCs and inhibit antigen-sensing by them. The expression of CD47 on TDEs inhibits their phagocytosis by immune cells and improves their retention in the circulation. Exosomal S100A9 downmodulates the maturation of DCs and decrease the expression of co-stimulatory CD83, CD86, IL-12 and IL-15 by DCs. Tumor exosomes induce DCs to express TGF-β, which further increases TGF-β expression in an autocrine loop, and robustly inhibits anti-tumor immunity. Higher levels of glycolytic enzymes detected on TDEs can impair DCs maturation by increasing ATP and lactate levels In TME

As a pivotal mechanism, DCs actively phagocyte tumor cells that have undergone immunogenic cell death, then process their antigens and present to T cells (priming their activation), but environmental sensing and phagocytosis, to some extent, are inhibited in tumors. For instance, it has been shown that the alarmin high mobility group protein B1 (HMGB1) recruits nucleic acids from dead tumor cells into DCs endosomes, leading to the innate sensing of tumors [51]. However, the T-cell immunoglobulin and mucin-domain containing-3 (TIM-3) highly expressed on tumor-infiltrating dendritic cells (TIDCs) interacts with the nuclear protein HMGB1 and suppresses nucleic acids sensing-mediated stimulation of DCs [51]. Tumors were also shown to secrete higher amount of exosome-bound TIM-3 and Galectin-9 (ligand for TIM-3) which can be bound to TIM3 receptors on the TIDC and interfere with the antigen recognition, while may also induce a cascade of inhibitory signals [52]. Based on a research, exosomes isolated from NSCLC patients have exhibited higher content of Galectin-9 compared to the exosomes from healthy control donors [52]. Likewise, the exosomes isolated from the cerebrospinal fluid (CSF) of the patients with glioblastoma multiforme (GBM) have also been shown to contain higher amounts of Galectin-9 [53]. It was demonstrated that the Galectin-9 on the surface of GBM-CSF-derived exosomes can interact with the TIM3 receptor on dendritic cells (DCs) in the CSF to inhibit antigen recognition, processing and presentation by these cells, resulting in the failure of the cytotoxic T-cell-mediated antitumor immune responses [53]. Therefore, tumor-derived exosomal Galectin-9 acts as a major regulator of tumor progression by inhibiting DCs maturation and antigen presentation to activate cytotoxic T-cells in the CSF and that loss of this inhibitory effect can lead to durable systemic antitumor immunity [53]. As mentioned, TDEs also harbor TIM-3, but it is not clear whether the exosomal TIM-3 can bound HMGB1 and interfere with nucleic acid sensing of DCs or not (Fig. 2).

The CD47 a “don’t eat me” signal, is another factor widely expressed by tumors which inhibits the sensing of mitochondrial DNA released by cancer cells via interaction with signal-regulatory protein-α (SIRPα) on DCs [18, 54]. By engaging SIRPα, CD47 limits the ability of DCs and macrophages to engulf tumor cells, which acts as a major phagocytic barrier [55]. The CD47 was also detected on the surface of exosomes released by tumors and the mouse mammary carcinoma-induced MDSCs, and was correlated with the enhanced retention of exosomes in the circulation [56, 57]. It has been suggested that the CD47 expression can protect TDEs from phagocytosis by monocytes and macrophages [58]. This was proven, since CD47 deprived exosomes exhibited significantly less retention, suggesting that CD47 presence on exosomes limits their clearance by circulating SIRPα+CD11+ monocytes [59]. It seems that by expressing CD47, TDEs may avoid to be taken up by DCs, but still can efficiently deliver their pro-tumorigenic contents. Exosomal CD47 has also been proven to facilitate MDSCs chemotaxis and migration, and accumulation of MDSCs in TME can further impair DCs maturation [60–62]. In light of these findings, TDEs harboring CD47 are assumed to play crucial roles in the tumor escape from immune cells (Fig. 2).

Others have also shown that TDEs enriched in S100A9 molecules are also capable of inhibiting DCs maturation [63]. A recent study revealed that exosomes isolated from afferent lymphatic fluid in patients with primary cutaneous melanoma have higher levels of S100A9 [63]. Immunohistochemistry and immunogold electron microscopy results confirmed the trafficking of tumor-derived S100A9 containing exosomes along the lymphatic path [63]. It was observed that the accumulation of S100A9 positive exosomes in the first node draining from the primary tumor, sentinel lymph node (SLN), is closely associated with a dysfunctional immune profile including reduced expression of dendritic cell maturation markers [63]. Importantly, this phenotype was observed prior to evidence of nodal metastasis [63]. These findings led to the conclusion that TDEs cargo, such as S100A9, may serve as early mediator of tumor-induced immune subversion in regional lymph nodes, establishing the niche for metastatic outgrowth. Likewise, others have also suggested that melanoma-derived extracellular vesicles (EVs) may participate in the premetastatic niche formation through cargo-specific polarization of DCs [50]. Accordingly, it was found that DCs matured in vitro in the presence of melanoma EVs had significantly impaired expression of CD83 and CD86 as well as decreased expression of Th1 polarizing chemokines Flt3L and IL15, and migration chemokines MIP-1α and MIP-1β compared to liposome-treated DCs [50]. Profiling of melanoma EV cargo revealed shared proteomic and RNA signatures including S100A8 and S100A9 protein cargo [50]. Further experiments showed that similar to melanoma EVs-treated DCs, the incubation of DCs with S100A8 and S100A9 proteins compromised their maturation in vitro. These findings suggest a role for S100A8 and S100A9 molecules in TDEs-mediated DCs abnormalities. These are in agreement with the earlier studies indicating that the higher level of S100A9 in the TME is, in part, responsible for the tumor-associated dendritic cells (TADCs)-mediated chemoresistance of breast cancer [64]. There is also other evidence indicating the importance of exosomal S100A9 in the altered maturation of DCs. In this regard, it has been demonstrated that paclitaxel can restore the maturation of DCs by decreasing the production of S100A9 and TNF-α by MDSCs, as the major source of the soluble/exosomal S100A9 in TME [65, 66]. In addition, exosomes enriched in S100A9 were also isolated from G-MDSCs and CLL patients, and were shown to induce the stemness of colorectal cancer cells by activating the NF-κB pathway [67]. All these findings indicate that tumor exosomes containing S100A8 and S100A9 proteins suppresses DCs maturation and improves the premetastatic niche formation in tumor-draining lymph nodes (Fig. 2).

In addition, previous studies have shown that treatment with tumor exosomes can induce TGF-b1 production in DCs [9, 34, 68–72]. Interestingly, this phenotype was associated with decreased expression of MHC class II and CD86 molecules, suggesting that TDEs inhibit the maturation of DCs [9]. TGF-b1 is known to inhibit the activation of lymphocytes and DCs, while converting effector T cells into Treg cells [73]. Moreover, exosomal TGF-β has also been proven to be essential for the cancer cell migration [74].

Along with their effect on DCs differentiation, glycolytic metabolites in the TME can also impact their maturation. Previously, several studies have shown that tumor-derived lactate renders human monocytes into less mature DCs that are deficient in IL-12 secretion and are not able to effectively stimulate T cells [23, 44]. As mentioned earlier, glycolytic enzymes have been identified in TDEs in substantial levels, which primarily convert extracellular glucose into ATP [42]. This was clearly mirrored by the tumor interstitial levels of ATP, which was demonstrated to be about 1000 times higher than those of normal tissues [75]. Since the presence of lactate dehydrogenase that catalyzes the conversion of pyruvate to lactate has been evidenced in TDEs, thus it is assumed that TDEs contribute to increased levels of lactate in the TME [43]. Eventually, these high levels of lactate can restrain DCs maturation while promoting the expansion of myeloid-derived suppressor cells (MDSCs), which are critically important for tumor progression [45]. There are several other studies have also confirmed that exosome-mediated metabolic reprogramming plays a crucial role in the intercellular communication between cancer cells and tumor associated cells. In this regard, it has been identified that tumor-associated macrophages (TAMs)-derived exosomes transfer HISLA to breast cancer cells, to prevent HIF-1a degradation, thus promoting aerobic glycolysis [76]. Instead, tumor cells release lactate that increases the expression of HIF-1α-stabilizing long noncoding RNA (HISLA) in TAMs [76]. All these findings highlight the importance of TDEs in metabolic reprogramming of TME, contributing to immune escape and tumor progression (Fig. 2).

### Tumor-derived exosomes alter DCs function

In addition to subverting DCs biology by altering differentiation (inducing toward MDSCs) and maturation (preventing acquisition of mature DCs features), tumors also interfere with the function (antigen-presenting capability) of fully matured DCs [77]. Notably, in early-stage tumors, DCs represent an immature phenotype which can induce paramount T cell proliferation ex vivo after being pulsed with tumor lysates, however at advanced stages, DCs are not simply immature and exhibit a semi-mature phenotype with compromised antigen-presenting activities [78, 79]. Indeed, DCs in advanced tumors exhibit a lower but still significant expression of MHC-II and costimulatory CD40; however, they also coexpress higher levels of co-inhibitory molecules (e.g. B7-H1) and exhibit increased arginase I and IDO activity comparable to that seen in MDSCs [21, 77]. Such DCs, called regulatory DCs, can result in either T cell anergy (unresponsiveness at the time of priming) or exhaustion (insufficient responses due to exposure to the negative costimulation), hence actively contribute to tumor growth through the inhibition of protective anti-cancer immunity [21]. How tumors induce immunosuppressive DCs has not clearly been identified, but there are multiple factors in TME that can transform conventional DCs with antigen-presenting capabilities into immunosuppressive players. Recent evidence indicates a significant role for tumor-derived exosomes (TDE) in altering the function of tumor-associated DCs [16]. Here, we reviewed the literature to gather findings on the importance of TDEs in impairing the function of DCs in the tumor context.

As mentioned, the lesser expression of MHC molecules on DCs in tumor bearing hosts has been assumed to considerably responsible for their compromised function. A recent study profiled the immune cells of the patients with pancreatic cancer has revealed substantial phenotypic changes in various immune cell populations, especially an increased population of immunosuppressive monocytes (CD14 + HLA-DRlo/neg) [31, 80, 81]. Further in vitro assessments demonstrated that the interactions between pancreatic TDEs and monocytes are responsible for HLA-DR downregulation in these cells [80]. Based on the observations, treatment of monocytes with TDEs can alter the STAT3 signaling pathway, which results in HLA-DR downregulation and upregulation of immunosuppressive arginase-1 expression and reactive oxygen species production [31, 80].

**Fig. 3:**
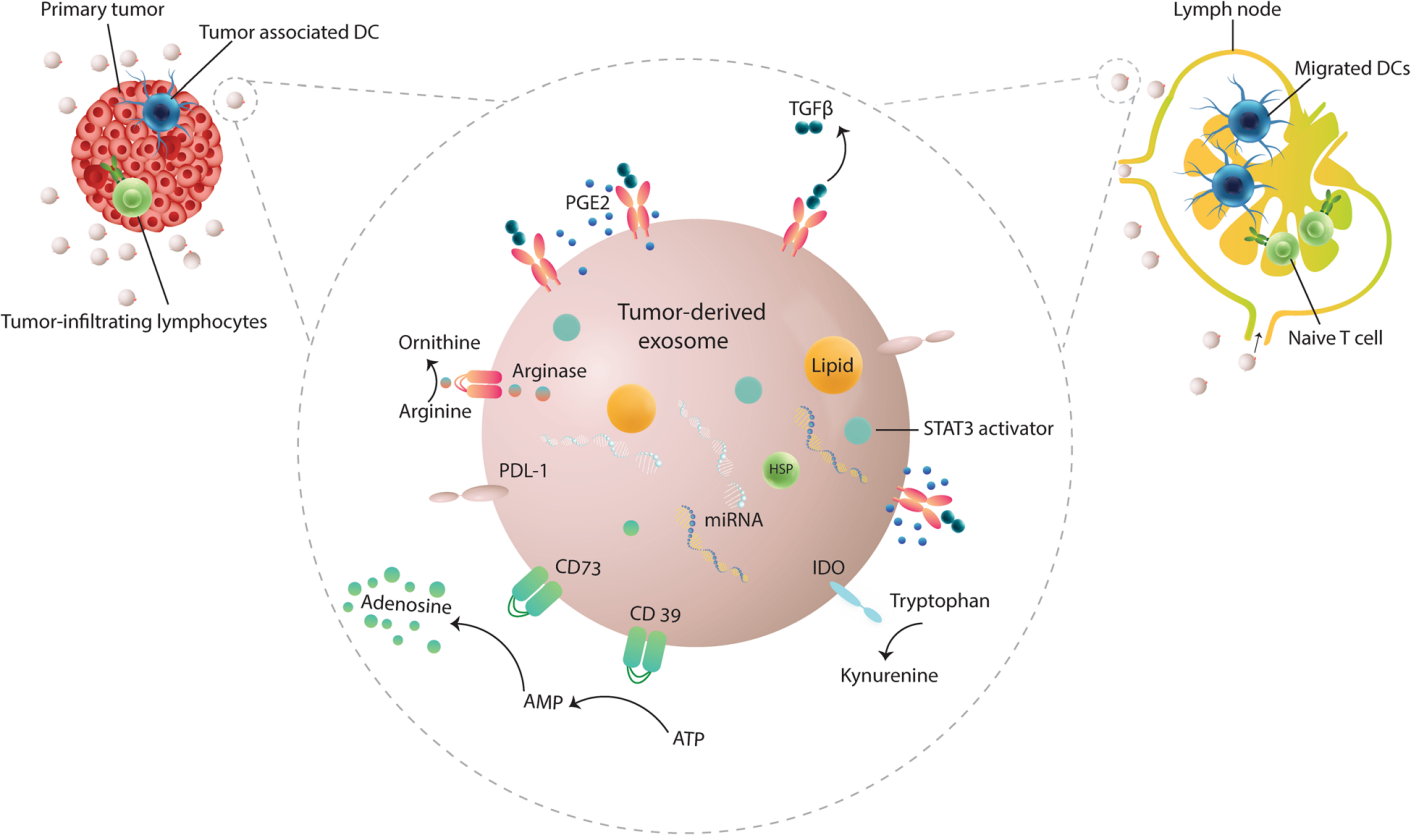
Tumor-derived exosomes inhibit normal function of dendritic cells. A plethora of inhibitory molecules including PD-L1, CD73, IDO (Indoleamine 2, 3-dioxygenase), L-arginase, PGE2, TGF-β, Lipids, and components of the STAT3 activators is presented in TDEs can reprogram DCs into immunosuppressive players and subvert their function either in priming or sustaining of anti-tumor immune responses. Exosomal PD-L1 interacts with PD-1 expressed on immune cells, including DCs and inhibits their function. IDO and L- arginase degrades tryptophan and arginine, respectively and thereby impedes effective priming of T cells. PGE2 and TGF- β are two inhibitory molecules enriched in TDEs which can impair antigen-presentation activity of DCs. Lipids and the STAT3 activating components can also be transported by TDEs, inducing dysfunctional DCs

In another study, it was found that GBM-derived extracellular vesicles do not directly inhibit T cell activation [82]. Rather than, these tumor-derived EVs induce immunosuppressive monocytes, thereby inhibit the activation of anti-tumor T cells [82]. The expression of PD-L1 on tumor-derived EV has been suggested to induce this inhibitory phenotype in monocytes [82]. Since tumor-associated DCs highly express PD-1, therefore PD-L1 expressing TDEs may negatively affect their function via PD-L1/PD-1 axis [83–85]. Likewise, several other studies have also shown that the exosomal PD-L1 can directly skew the function of immune cells toward tumor-promoting phenotype [86]. In another study, it was found that treatment of DCs with TDEs significantly inhibited the maturation and migration of DCs [12]. These TDEs-treated DCs drastically decreased CD4 + IFN-γ + Th1 differentiation but increased the rates of regulatory T (Tregs) cells. Further experiments revealed that the immunosuppressive ability of tumor exosome-treated DCs was partially restored with PD-L1 blockade [12]. The most recent studies indicate that exosomal PD-L1 plays a vital role in tumor immune escape as well as in tumor resistant to anti-PD-1/PD-L1 immunotherapy [12] (Fig. 3). Besides of its expression on TDEs, it has also been shown that TDEs can induce PD-L1 expression on monocytes, the precursor to DCs and macrophages [87, 88]. In this regard, exosomes from glioblastoma (GBM)-derived stem cells (GSCs) were shown to traverse the monocyte cytoplasm, causing a reorganization of the actin cytoskeleton, and skew monocytes toward the immunosuppressive M2 phenotype, including programmed death-ligand 1 (PD-L1) expression [87]. Mass spectrometry analysis demonstrated that the GDEs contain a variety of components, including members of the signal transducer and activator of transcription 3 (STAT3) pathways that functionally mediate this immunosuppressive switch [87]. Western blot analysis revealed that upregulation of PD-L1 in GSC exosome-treated monocytes and GBM-patient-infiltrating CD14+ cells predominantly correlates with increased phosphorylation of STAT3 [87]. Others have shown that the paired expression of PD-1; PD-L1 on DCs is correlated with the tumor progression, loss of positive costimulatory markers (CD80, CD86, and CD40), a lack of cytokine release (IL-12, IL-10, IL-6, TNFα, and G-CSF), and contact-dependent inhibition of T cell expansion [78, 89]. Cumulatively, these data indicate that TDEs are potent modulators of the tumor-associated immunosuppressive microenvironment and play a significant role in DCs functional abnormalities (Fig. 3).

Tumor-derived exosomes may also contribute to DCs dysfunction through indoleamine-pyrrole 2, 3-dioxygenase (IDO) pathway [90–92]. In a previous study, DCs cultured with IDO+ exosomes derived from BMSCs had downregulated CD40, CD86, CD80, MHC-II, but the increased secretion of anti-inflammatory cytokines compared with the other groups [93]. It has been shown that tumor/exosomal IDO produces kynurenine by degrading tryptophan, which in turn can induce IDO activity in DCs by interacting with the aryl hydrocarbon receptor (AHR) [94, 95]. This is in line with the previous studies indicating that tumor-associated immunosuppressive DCs are the major source of IDO within the tumor-microenvironment promoting malignant progression [96]. Additionally, functionally compromised DCs can also release IDO+ exosomes, which may further enhance immunosuppression [97]. Therefore, tumor exosomes carrying IDO can contribute to DCs dysfunction by producing kynurenine as well as inducing the expression of IDO on DCs (Fig. 3). In addition, arginase-1 (ARG1), another key enzyme driving immunosuppression, was also detected in exosomes from several cancers [98–100]. Recently, it has been found that exosomes isolated from the ascites and plasma of ovarian cancer patients contain ARG1 [99]. The findings demonstrated that ARG1-containing exosomes are transported to draining lymph nodes and taken up by dendritic cells, leading to the inhibition of antigen-specific T-cell proliferation. It is well known that the upregulation of ARG1 activity in TME results in a reduced availability of arginine [101]. Previous studies clearly show that drops in the extracellular arginine levels can induce DCs dysfunction via downregulating the MHC-II molecules [102, 103]. Tumor exosomes and arginine restriction might also induce ARG1 expression on DCs, further enhancing immunosuppression [24, 99]. This is in agreement with the previous reports showing that DCs isolated from advanced tumors exhibit significant L-arginase activity [79]. Besides, exosomal ARG1 can also directly inhibit immune responses, since arginine is essentially needed for the activity of effector T cells [99].

It has been shown that PGE2 and TGF-β, both present in TDEs, are also critically involved in the abrogated function of tumor-associated DCs via the upregulation of ARG1 activity, IDO, and co-inhibitory molecule B7-H1 and B7-DC, as well as the IL-10 production [33, 104]. In addition, exosomal PGE2 and TGF-β were clearly demonstrated to inhibit DCs function through the induction of tolerogenic mediators, two ecto-enzymes CD39 and CD73, that act sequentially to generate anti-inflammatory extracellular adenosine [16]. In a recent study, it was found that exosomes derived from prostate cancer cells contain PGE2 which can induce CD73 expression on DCs and suppress their function [16]. CD73 was proven to pair with CD39 that is consistently expressed on DCs, and converts extracellular ATP into adenosine. The subsequent engagement of adenosine with the adenosine A2A receptor (A2AR), expressed on DCs and effectors T cells, could robustly play against anti-tumor immunity [105] (Fig. 3).

Other tumor-microenvironment components can also impair tumor-associated antigen presentation capability of DCs. For instance, the higher levels of lipid peroxidation can increase endoplasmic reticulum stress of DCs in tumor-microenvironment, which in turn impair the function DCs by increasing lipid accumulation [106]. Indeed, it has been shown that DCs with a higher load of lipids have the defective ability in processing and cross-presentation of exogenous antigens [106, 107]. Moreover, the intracellular lipid accumulation can inhibit the effective trafficking of MHC-I-peptide complexes to the cell surface [106, 108]. Recently, it has been identified that TDEs contribute to DCs dysfunction by transferring fatty acids [109]. Based on the evidence, delivering fatty acids by TDEs could induce the expression of peroxisome proliferator-activated receptor (PPAR) in DCs, which in turn increase both the biogenesis and oxidation of fatty acids [109]. The excess amount of intracellular lipid droplets/ fatty acid oxidation-by products can result in dysfunctional DCs via increased mitochondrial oxidative phosphorylation [109]. Therefore, based on these findings, TDEs can induce metabolic reprogramming in DCs either by transferring or inducing the production of lipids (Table 1 and Fig. 3).

**Table 1:** The content of tumor-derived exosomes (TDEs) and their effects on developmental stages of DCs

Exosome content	Mechanism of Action	Ref
**Inhibition of DCs Differentiation**		
Cox-2, PGE2, TGF-b1, IL-6, HSP-70, and HSP-72	Promoting the polarization of myeloid-derived suppressor cells (MDSCs), mainly through the STAT-3 pathway	[24–29, 31–33, 35–38]
Glycolytic Enzymes	Increasing ATP and lactic acid levels and enhancing MDSCs population	[18, 42–45]
HLA-G	Blocking monocyte-derived DCs differentiation	[39]
**Inhibition of DCs Maturation**		
Galectin-9 and TIM-3	Interacting with TIM-3 on DCs and reducing nucleic acid sensing	[51–53]
CD-47	Reducing phagocytosis by interacting with SIRP-a on DCs	[54–60]
S100A9	Downregulating CD83, CD86, IL-12 and IL-15 expression levels	[63–67]
TGF-b1	Induction of TGF-b1 secretion by DCs	[9, 68, 73, 74]
Lactate dehydrogenase	Increasing ATP and lactate levels in tumor microenvironment	[43–45, 75, 76]
**Inhibition of DCs Function**		
STAT3 activators	Reducing the levels of MHC and CD83 and CD86 molecules	[31, 34, 80]
PD-L1	Inducing PD-1 expression and transferring of negative signals	[12, 82–88]
IDO	- Decreasing the levels of CD40, CD83, CD86 and MHC molecules - Degrading tryptophan into kynurenine - Kynurenine-meditated increase of IDO expression on DCs	[93, 95–97]
L-arginase (ARG1)	-Impedes the DCs-mediated T cells priming in regional lymph nodes - Reduces arginine level in tumor microenvironment, resulting in lower expression of MHC molecules	[24, 98–100]
PGE and TGF-b1	Increasing CD73 expression on DCs, resulting in increased levels of inhibitory adenosine in tumor site	[16, 24, 104, 105]
Lipids	Accumulating lipids in DCs, interfering with their antigen-presentation function	[106–109]

### Lessons learned from TDEs-mediated DCs dysfunction

In spite of containing a variety of immunosuppressive biomolecules, TDEs are also rich in tumor antigens and could provoke anti-tumor immunity [110]. Previously, it has been demonstrated that DCs could uptake TDEs, process their antigens and present to CD4 and CD8 positive T cells via MHCII and MHCI, respectively, inducing antigen-specific CTL responses [110]. These findings inspired numerous studies to investigate the potential utility of TDEs (isolated from patients’ plasma or tumor cell cultures) as tumor antigen sources in DC-based vaccination for cancer prevention and treatment [15, 110, 111]. There is now a great deal of evidence that shows greater anti-tumor activity for TDEs-pulsed DCs in comparison to tumor lysate-loaded DCs, giving rise to a consensus that DCs loaded with TDEs could serve as a novel promising approach for tumor immunotherapy [112]. However, the immunoinhibitory content of TDEs that causes DCs to become dysfunctional, as discussed in this review, has largely been overlooked in TDEs-loaded DC vaccine strategies [14]. It might be expected that immunosuppressive cargo of TDEs would affect the therapeutic potential of TDEs-loaded DCs. This idea is supported by the findings showing that the engineered exosomes lacking inhibitory molecules can induce more effective anti-tumor responses in DC-based vaccine design [113, 114]. For instance, it has been demonstrated that DCs loaded with TGF-b1-depleted exosomes induce greater anti-tumor CTLs compared to DCs pulsed with TGF-b1-expressing exosomes [113, 115]. In another study, it was also found that treatment of DCs with TDEs loaded with interleukin 12 (IL-12) or deprived of TGF-b1 could strongly support induction of anti-tumor immune responses compared to unmodified TDEs [116]. This shows that engineering exosomes to carry a customized cargo can be helpful in maximizing the therapeutic benefits of TDEs-loaded DC vaccines and should be carefully considered in future studies [114].

**Fig. 4:**
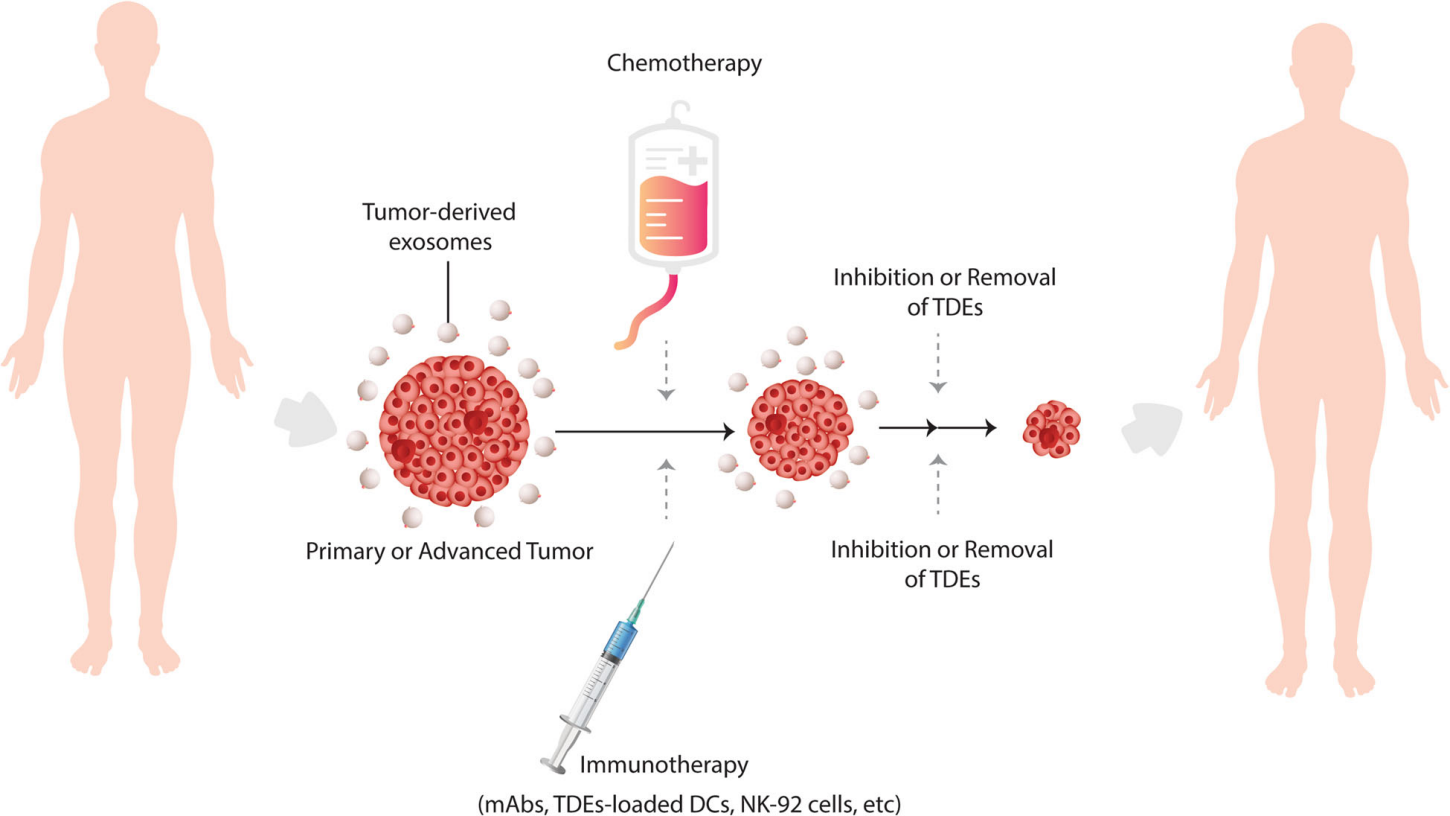
Combining targeted tumor exosome inhibition or removal with exiting chemo- and immunotherapies. Tumor exosomes induce resistance to chemotherapies and counteract beneficial effects of immunotherapies including monoclonal antibodies (mAbs), adoptive transfer of NK-92 cells and possibly TDE-loaded DCs. Adjunctive inhibition or removal of TDEs may add to the therapeutic benefits of currently available chemo- and immunotherapies and could improve tumor regression

Furthermore, since tumors constantly release exosomes into the surrounding environment as well as into the circulation, these virus-sized vesicles are very likely to also interfere with the immune therapies in vivo, including DC vaccines [117, 118]. This becomes more evident, as the immunosuppressive cargo of TDEs has been evidenced to abolish the efficacy of adaptive NK92 cell therapy in acute myeloid leukemia patients [117]. Circulating TDEs have also been proved to interfere with the therapeutic effects of monoclonal anti-HER2, −CD20 and -PD-1/PD-L1 antibodies [119–123]. Moreover, tumor exosomes have widely been reported to mediate resistance to common chemotherapies [86, 117, 124–126]. However, strikingly, targeting exosomal inhibitory biomolecules or blockade of exosome release from cancer cells could strongly induce anti-tumor immunity and improve the anti-cancer effects of chemotherapeutic agents [118, 120, 127–130]. These data suggest that a strategy for targeting circulating tumor exosomes could add to the benefits of chemo- and immunotherapeutic interventions, possibly including DC-based therapies [118] (Fig. 4).

### Advances in targetting tumor-derived exosomes

Due to the pivotal role that TDEs play in multiple aspects of tumor development and growth, such as proliferation, angiogenesis, metastatic niche formation and immune escape, a strong interest has emerged in recent years to selectively inhibit the generation/release of tumor exosomes as an adjunctive therapy for cancer [120, 127, 128]. The early research on exosome formation showed that these particles are highly enriched in sphingolipid ceramide and their release is significantly reduced in the presence of GW4869, a small molecule that inhibits neutral sphingomyelinase2 (nSMase2) [131, 132]. Further studies demonstrated that in addition to nSMase, ras-related RAB proteins are also important players of exosome biogenesis and knocking-down of RAB27A and RAB27B could significantly inhibit exosome shedding [133–135]. These findings provided preliminary insights into the underlying mechanisms of exosomes generation and unveiled potential targets for inhibiting their release. Over the past decade, tremendous efforts have been devoted to explore compounds capable of inhibiting nSMase and RAB27A expression as a possible route to block exosome secretion [135]. As a result, manumycin A [136], spiroepoxide [137, 138], cambinol [139], scyphostatin [140, 141], and DPTIP [142] were found to decrease exosome production by downregulating nSMase expression. More recently, researchers have used a high-throughput screening (HTS) technique to identify currently exited compounds with drug repositioning potential for exosome inhibition [143]. A total number of 4580 pharmacologically active compounds from the LOPAC library and the NPC library were examined and only tipifarnib, neticonazole, climbazole, ketoconazole, nexinhib20, nexinhib4, were found as potent exosome inhibitors [143]. Among these compounds, it has been demonstrated that nexinhib20 and nexinhib4, inhibitors of neutrophil exocytosis, can suppress exosome biogenesis by selective inhibition of RAB27A [144], however, tipifarnib, neticonazole, climbazole, and ketoconazole were shown to decrease exosome secretion by inhibiting RAB27A, Alix and nSMase2 [143]. Of note, the therapeutic value of tipifarnib in the adjuvant setting is under investigation in several clinical trials, and ketoconazole has currently been approved for the treatment of prostate cancer patients by the US Food and Drug Administration (FDA) [145–148]. Several other currently available drugs have also been identified with potential exosome inhibiting effects. Sulphisoxazole [149], ketotifen [150], cannabidiol [151, 152], pantoprazole [153, 154], esomeprazole [154], and imipramine [155] have been reported to exert potent blocking effects on exosome production with anti-cancer activity. Others have shown that chloramidine [155], bisindolylmaleimide-I [155], and the vitamin B5 derivative pantethine [156] can also inhibit the secretion of tumor exosomes. Dasitinib, a dual BCR/ABL and Src family tyrosine kinase inhibitor, was shown to prevent exosome release while promoting apoptosis in K562R (IMT) cells [157]. Recently, a synthetic peptide derived from the secretion modification region (SMR) of HIV-1 Nef, which carried PEG on the N-terminus and a Clusterin (Clu)-binding peptide on the C-terminus, was reported to inhibit metastasis and angiogenesis by causing a decrease in exosome release [158]. WEB2086, an antagonist of platelet-activating factor receptor (PAFR), was also proven to inhibit exosome release [159]. Dimethyl amiloride, a drug used to treat high blood pressure, has also been reported to inhibit exosome formation [36]. Additionally, anti-CD9 and anti CD63 antibodies as well as a hemofiltration device known as the Aethlon ADAPT™ (adaptive dialysis-like affinity platform technology) were shown to be useful in removing exosomes from circulation [130, 160].

By advances in our understanding of the basic biology of exosome formation and release, a number of new targets have also been identified. It has been shown that the gene silencing of tumor susceptibility gene 101 (TSG101), a member of Vps protein family which involves in exosome trafficking, inhibits exosome production in colon cancer cells [161]. Annexin A1 (ANXA1) has also been documented to play an important role in inward vesiculation and its suppression was associated with reduced exosome secretion in pancreatic cancer cells [162]. The proline-rich Akt substrate of 40 kDa (PRAS40) has also been reported to regulate exosome secretion in breast and lung cancer cells [163]. Others have shown that the blocking of protease-activated receptor (PAR)-2, which binds to the tissue factor/factor VIIa, suppresses the secretion of TF-positive exosomes from pancreatic cancer cells [164].

### Future perspectives and concluding remarks

The literature reviewed in this paper indicates that TDEs impair differentiation, maturation and function of DCs to favor immune escape and tumor outgrowth. Although several well-defined, proven mechanisms underlying the inhibitory effects of tumor exosomes on DCs biology were discussed in this review, but TDEs may also alter DCs behavior by a number of speculative mechanisms. For example, blockade of DCs differentiation has primarily been attributed to the presence of tumor-derived vascular endothelial growth factor (VEGF), and its levels were negatively correlated with the number of DCs in the circulation and TME in human cancers [18, 20, 21, 26, 165–172]. Tumor-derived exosomes were also shown to induce the release of VEGF by transferring miRNA-21 into recipient cells, thus leading to increased VEGF levels within the tumor [173–175]. More recent findings also show that TDEs harbor an active isoform of VEGF, which is associated with the tumor outgrowth and resistance to common monoclonal antibody (mAb) therapies [176]. Besides, tumor cells also secrete excessive amounts of the gangliosides GD2 and GM3 that inhibit the differentiation of DCs from CD34^+^ as well as monocytic precursors, and induce apoptosis of monocyte-derived DCs [177–179]. These sialic acid-containing glycosphingolipids were also shown to be shed from tumors via exosomes and can actively suppress immune cells [26, 180]. Therefore, it can be postulated that several other exosomal biomolecules, including but not limited to VEGF, miRNA-21 and gangliosides, might play a role in the impaired differentiation of DCs in tumor context; however, their role has yet to be investigated. In addition, tumor exosomes were also reported to contain notable amounts of IL-10 [33, 74, 81, 181, 182]. The high levels of IL-10 were shown to inhibit DCs maturation by downmodulating the expression of MHC-I and costimulatory molecules, blocking the initiation of T cell responses [7, 21, 183, 184]. However, there is no evidence thus far that IL-10 plays a role in TDEs-mediated DCs defects. Future studies can shed light on the link between the above-mentioned exosomal markers and DCs abnormalities in cancer. Additionally, since different subpopulations of DCs exhibit distinct phenotypic characteristics and functional potential, thus it will be very important in future research to focus more attention on the effects of TDEs on DCs subgroups. Also, as discussed later in this review, great efforts have been made so far to target exosomes or exosomal markers to inhibit tumor progression and improve anti-tumor immunity. However, regardless of significant progress has been made in recent years in the discovery of exosomes inhibitors; it is still in its infancy and the therapeutic value of those inhibitors as adjunctive therapy for cancer has not yet been fully validated. Most of the compounds tested for exosome inhibition were highly cytotoxic and did not show selectivity to inhibit tumor exosomes, and thus may disrupt intercellular communication by inhibiting exosome secretion from non-tumor cells, leading to unwanted adverse side effects. Therefore, there are still significant challenges ahead to identify novel compounds and viable targets for selective inhibition or removal of tumor exosomes. In summary, growing evidence supports the notion that tumor-derived exosomes are potential suppressors of immune cells, including DCs, and targeting these extracellular vesicles may provide a new avenue for the better treatment of cancers.

## Data Availability

Not applicable.

## Abbreviations

TDEs: Tumor-derived exosomes
mRNA: Messenger RNA
Fas-L: Fas ligand
PD-L1: Programmed death ligand1
NK cell: Natural killer cell
APCs: Antigen presenting cells
DCs: Dendritic cells
MDSCs: Myeloid-derived suppressor cells
pDCs: Plasmacytoid dendritic cells
cDCs: Conventional dendritic cells
Ag: Antigen
CD4: Cluster of differentiation 4
CD8: Cluster of differentiation 8
MHC-I: Major histocompatibility molecule 1
MHC-II: Major histocompatibility molecule 2
VEGF: Vascular endothelial growth factor
NF- κB: Nuclear factor kappaB
FLT3L: FMS-like tyrosine kinase 3 ligand
mAb: Monoclonal antibody
miRNA: MicroRNA
STAT3: Signal transducer and activator of transcription 3
PGE2: Prostaglandin E2
COX-1: Cyclooxygnase-1
COX-2: Cyclooxygenase 2
TGF-β: Transforming growth factor beta
IL-6: Interleukin 6
Hsp70: Heat shock protein 70
Hsp72: Heat shock protein 72
MyD88: Myeloid differentiation primary response 88
S100A8: S100 calcium-binding protein A8
S100A9: S100 calcium-binding protein A9
HLA-G: Human leukocyte antigen G
CSC: Cancer stem cell
ATP: Adenosine three phosphate
PAMPs: Pathogen-associated molecular patterns
DAMPs: Damage-associated molecular patterns
TNF-R: Tumor necrosis factor receptor
IL-1R: Interleukin 1 receptor
TLRs: Toll like receptors
CD40: Cluster of differentiation 40
CD80/86: Cluster of differentiation 80/86
BMDCs: Bone marrow-derived dendritic cells
HMGB1: High mobility group box 1 protein
TIM-3: T-cell immunoglobulin and mucin-domain containing-3
TIDCs: Tumor infiltrating dendritic cells
NSCLC: Non small-cell lung carcinoma
GBM: Glioblastoma multiforme
CSF: Cerebrospinal fluid
SIRPα: Signal regulatory protein α
SLN: Sentinel lymph node
EVs: Extracellular vesicles
Th1: T helper cell
IL-15: Interleukin 15
MIP-1α/ β: Macrophage inflammatory protein 1alpha/beta
TNF-α: Tumor necrosis factor alpha
HIF-1: Hypoxia-inducible factor 1
IL-10: Interleukin 10
TAMs: Tumor-associated macrophages
HISLA: HIF-1α-stabilizing long noncoding RNA
B7-H1: B7 homolog 1
IDO: Indoleamine 2, 3-dioxygenase
TME: Tumor microenvironment
HLA-DR: Human leukocyte antigen – DR isotype
IFN-γ: Interferon gamma
Tregs: Regulatory T cells
G-CSF: Granulocyte-colony stimulating factor
AHR: Aryl hydrocarbon receptor
A2AR: Adenosine A2A receptor
PPAR: Peroxisome proliferator-activated receptor
Rab27a: Ras-related GTPase 27a
nSMase2: Neutral sphingomyelinase 2
FDA: Food and Drug Administration
